# Evaluation of sleep disorders in children and adolescents affected by Klinefelter syndrome

**DOI:** 10.1007/s00431-024-05944-2

**Published:** 2025-01-11

**Authors:** Roberto Paparella, Fabiola Panvino, Luisiana Gambuti, Andrea Cerrito, Alessia Pallante, Ginevra Micangeli, Michela Menghi, Francesco Pisani, Oliviero Bruni, Ignazio Ardizzone, Luigi Tarani

**Affiliations:** 1 Department of Maternal Infantile and Urological Sciences, Sapienza University of Rome, Rome, Italy; 2 Department of Human Neuroscience, Section of Child and Adolescent Neuropsychiatry, Sapienza University of Rome, Rome, Italy; 3 Department of Women’s and Children’s Health, University of Padua, Padua, Italy; 4 Department of Developmental and Social Psychology, Sapienza University of Rome, Rome, Italy

**Keywords:** Klinefelter syndrome, Sleep disorders, Children, Adolescents, Questionnaire, SDSC

## Abstract

Klinefelter syndrome (KS) is the most common sex chromosomal aneuploidy in males (47,XXY karyotype in 80–90% of cases), primarily characterized by hypergonadotropic hypogonadism and infertility. It encompasses a broad phenotypic spectrum, leading to variability in neurocognitive and psychosocial outcomes among affected individuals. Despite the recognized correlation between KS and various neuropsychiatric conditions, studies investigating potential sleep disorders, particularly in pediatric subjects, are lacking. This study aimed to investigate the presence of sleep-related behaviors potentially suggestive of a sleep disorder in a cohort of pediatric patients with KS, in comparison with a group of healthy male age-matched controls. During the period from January to December 2023, a validated sleep questionnaire (Sleep Disturbance Scale for Children: SDSC) was administered to the primary caregivers of 80 children with KS: 40 of preschool age (3–5 years) and 40 of school age (6–16 years). Data were compared with a control group of 180 healthy age-matched male children: 90 of preschool age (3–5 years) and 90 of school age (6–16 years). Among preschoolers, the proportion of subjects with pathological non-restorative sleep T-scores was significantly higher in the KS group compared to controls (p = 0.03). In both KS and control groups, school-aged subjects had higher questionnaire scores compared to preschoolers. The school age KS group had significantly higher mean total T-scores and mean T-scores for disorders of initiating and maintaining sleep (DIMS), disorders of arousal (DA), and disorders of excessive somnolence (DOES) compared to controls (*p *< 0.01 for all). The KS group also showed significantly higher percentages of children with clinically relevant T-scores for DIMS, DA, DOES, sleep hyperhidrosis, and total T-scores.

*Conclusion*: Our study indicates that sleep disorders are more prevalent in children with KS than in the general population, especially in the school age group. Screening for sleep issues in the clinical setting using tools like the SDSC is warranted, and should start from age 6 for children with KS. Further research is needed to better understand the origins of these disturbances, the role of comorbidities, and their long-term effects to improve diagnosis and treatment strategies for these patients.

**Table:** 

**What is Known:**	
• *Neurocognitive and psychosocial disorders can be observed in individuals with KS.*	
• *Sleep disorders may be associated with various neuropsychiatric conditions; however, they have not been sufficiently explored in individuals with KS, particularly in pediatric populations.*	
**What is New:**	
• *Sleep-related problems are more common in children with KS compared to the general population, especially in the school age group with regard to DIMS, DA, and DOES factors.*	
• *Starting from 6 years of age, the SDSC might be a promising early diagnostic tool for sleep disorders in children with KS.*

## Introduction

Klinefelter syndrome (KS) is the most prevalent sex chromosome aneuploidy, with an estimated incidence rate of 1 in 500 to 660 male newborns [1]. Traditionally associated with hypogonadism and infertility [2], the syndrome is accompanied by a diverse array of clinical abnormalities ranging from metabolic syndrome to cardiovascular, endocrine, and neuropsychopathological morbidities [3–5]. Despite extensive research into the medical and psychological aspects of KS, limited attention has been paid to sleep disturbances, particularly during childhood and adolescence. Recent studies have started to explore its broader implications, including its potential influence on sleep patterns in affected individuals, though primarily in adults [6–8].

The impact of sleep disorders on children is substantial, as these disturbances are often linked to various behavioral and cognitive problems, further exacerbating the challenges faced by children with KS [9]. Several studies have demonstrated that sleep disturbances are more prevalent among children with chronic health conditions, such as neurodevelopmental and metabolic disorders [10, 11], but the specific relationship between KS and sleep has not been systematically explored. Factors such as neurological and endocrinological anomalies, which are common in KS, are well-known to contribute to poor sleep quality in the general population [12], yet little is known about how these factors specifically affect sleep in patients with KS.

The existing body of literature lacks comprehensive coverage concerning sleep disturbances in KS, particularly in pediatric subjects, despite the well-established correlation between such disorders and neuropsychiatric conditions—possible comorbidities of the syndrome [13]. Moreover, the recently published European Academy of Andrology guidelines on KS do not encompass a specific examination or evaluation of sleep-related behaviors or disorders [14]. To fill this knowledge gap, our study aimed to investigate sleep-related behaviors in pediatric patients with KS compared to healthy age-matched male controls.

## Materials and methods

### Study population

The study was conducted between January and December 2023 and included 80 children with KS recruited from the Department of Maternal Infantile and Urological Sciences, Sapienza University—Policlinico Umberto I, Rome, Italy. Participants were identified during regular follow-up visits or through organizations supporting individuals with KS and their families. The inclusion criteria were as follows: (1) prenatal diagnosis of classic, non-mosaic 47,XXY KS, confirmed by postnatal chromosome analysis on peripheral blood; (2) age 3–16 years. The exclusion criteria were as follows: (1) presence of other known genetic or chromosomal disorders; (2) current or prior testosterone replacement therapy; (3) overweight or obesity. The control group consisted of 180 healthy, non-overweight/non-obese male children aged 3–16 years, recruited from the Child Neuropsychiatry Institute of Sapienza University—Policlinico Umberto I, Rome, Italy. None had diagnosed neuropsychiatric conditions, as confirmed by comprehensive clinical evaluations, nor any known genetic or chromosomal disorders. Additionally, none were receiving psychoactive medications or treatments that could influence sleep patterns.

For subgroup analyses, participants in both KS and control groups were divided by age:

- preschool age (3–5 years): 40 patients (mean ± standard deviation (SD): 3.8 ± 0.8), 90 controls (mean ± SD: 4.0 ± 0.8);

- school age (6–16 years): 40 patients (mean ± SD: 10.8 ± 4.0), 90 controls (mean ± SD: 10.0 ± 2.6).

### Sleep Evaluation

Primary caregivers of children in both groups were administered a validated questionnaire, the Sleep Disturbance Scale for Children (SDSC), for the evaluation of sleep-related behaviors potentially indicative of a sleep disorder [15, 16], with the questionnaires being completed by the children's mothers in all cases. The SDSC investigates sleep behavior and disorders over the preceding six months (a period of six months was chosen in the original study due to the low frequency in the population of some sleep disorders and to better discriminate between transient and persistent disorders). It consists of 26 items on a Likert-type scale ranging from 1 to 5, with higher scores indicating greater clinical severity of symptoms.

Items are divided into six categories representing some of the most common sleep difficulties affecting adolescents and children. Regarding school-aged patients [15], the factors are as follows:

1. Disorders of initiating and maintaining sleep (DIMS).
2. Sleep breathing disorders (SBD).
3. Disorders of arousal/nightmares (DA).
4. Sleep–wake transition disorders (SWTD).
5. Disorders of excessive somnolence (DOES).
6. Sleep hyperhidrosis (SHY).

The SDSC was also validated for use in preschool age, with a distinct structure compared to the original questionnaire, given the different prevalence of sleep disturbances in younger children, but with a similar total cut-off score. Regarding preschool-aged patients [16], the factors are as follows:

1. Parasomnias (PAR).
2. Disorders of initiating and maintaining sleep (DIMS).
3. Sleep breathing disorders (SBD).
4. Disorders of excessive somnolence (DOES).
5. Sleep hyperhidrosis (SHY).
6. Non-restorative sleep (NRS).

The total score is derived by adding up the scores for each of the 26 items, with a potential range between 26 and 130. Both the score for each of the six individual factors and the total score are converted into a T-score: a T-score > 70 for both school and preschool age is considered “pathological”, while a T-score between 55 and 70 for school age or between 61 and 70 for preschool age is considered “suspect/borderline” [15, 16].

### Statistical analysis

The analysis of mean differences in total and individual factor T-scores between subjects with KS and controls, across both age groups, was performed using the Mann–Whitney U test for non-parametric data. Prior to this, the Shapiro–Wilk test was conducted to assess the normality of the data, revealing a non-normal distribution. The effect size for the Mann–Whitney U test was calculated using rank-biserial correlation (*r*), interpreted as follows: *r* < 0.10 = very small, 0.10–0.29 = small, 0.30–0.49 = moderate, and ≥ 0.50 = large [17]. The statistical software Jamovi (version 2.3.28.0) was used for these analyses.

To compare the percentage of subjects with clinically relevant T-scores (total and individual factor T-scores) between the KS and control groups in preschool and school-age groups, the Chi-square test was applied. For the Chi-square test, Cramer’s V was used as the effect size measure, with the following interpretation: V > 0.25 = very strong, 0.15–0.25 = strong, 0.10–0.15 = moderate, 0.05–0.10 = weak, and 0–0.05 = no or very weak association [18]. Microsoft Excel software was used for statistical analyses.

Statistical significance was set at p ≤ 0.05, with results meeting this threshold considered statistically significant.

## Results

### Preschool age

With the exception of the SBD factor, mean T-scores were higher in the KS group versus the control group. A significant difference with a small effect size (p = 0.01, *r* = 0.28) was observed with regard to the PAR factor (Table 1).

**Table 1:** Preschool age: differences in total and individual factor T-scores between KS group and control group

	T-score (mean ± standard deviation)		p-value	Effect size
	Cases	Controls		
**Parasomnias**	53.8 ± 9.3	50.0 ± 9.0	0.01 *	0.28
**Disorders of initiating and maintaining sleep**	51.3 ± 11.1	50.3 ± 9.6	0.74	0.04
**Sleep breathing disorders**	47.8 ± 9.7	49.9 ± 11.2	0.13	0.16
**Disorders of excessive somnolence**	51.5 ± 9.2	48.9 ± 7.7	0.10	0.15
**Sleep hyperhidrosis**	50.3 ± 7.8	50.1 ± 9.6	0.58	0.06
**Non-restorative sleep**	54.7 ± 12.5	51.1 ± 8.6	0.23	0.13
**TOTAL**	53.2 ± 10.3	50.6 ± 9.2	0.13	0.17

*Statistically significant (p ≤ 0.05). For the Mann–Whitney test, effect size is given by the rank biserial correlation (*r*)

Analysis of the percentages of patients with clinically significant T-scores (total and individual factors) revealed no statistically significant differences between the KS and control groups, except for a higher proportion of pathological NRS scores in the KS group (p = 0.03, V = 0.19) (Table 2).

**Table 2:** Preschool age: percentages of subjects with clinically relevant total and individual factor T-scores

		Percentage (n/total)		χ^2^	p-value	V
		Cases	Controls			
**Parasomnias**	Borderline Pathological Overall altered	10% (4/40) 10% (4/40) 20% (8/40)	7.8% (7/90) 3.3% (3/90) 11.1% (10/90)	0.18 2.42 1.80	0.67 0.12 0.18	0.04 0.14 0.12
**Disorders of initiating and maintaining sleep**	Borderline Pathological Overall altered	5% (2/40) 10% (4/40) 15% (6/40)	5.6% (5/90) 7.8% (7/90) 13.3% (12/90)	0.02 0.18 0.06	0.90 0.67 0.80	0.01 0.04 0.02
**Sleep breathing disorders**	Borderline Pathological Overall altered	5% (2/40) 7.5% (3/40) 12.5% (5/40)	6.7% (6/90) 6.7% (6/90) 13.3% (12/90)	0.13 0.03 0.02	0.72 0.86 0.90	0.03 0.02 0.01
**Disorders of excessive somnolence**	Borderline Pathological Overall altered	7.5% (3/40) 5% (2/40) 12.5% (5/40)	6.7% (6/90) 1.1% (1/90) 7.8% (7/90)	0.03 1.85 0.73	0.86 0.17 0.39	0.02 0.12 0.08
**Sleep hyperhidrosis**	Borderline Pathological Overall altered	10% (4/40) 2.5% (1/40) 12.5% (5/40)	12.2% (11/90) 6.7% (6/90) 18.9% (17/90)	0.14 0.95 0.81	0.71 0.33 0.37	0.03 0.09 0.08
**Non-restorative sleep**	Borderline Pathological Overall altered	5% (2/40) 17.5% (7/40) 22.5% (9/40)	5.6% (5/90) 5.6% (5/90) 11.1% (10/90)	0.02 4.75 2.81	0.90 0.03 * 0.09	0.01 0.19 0.15
**TOTAL**	Borderline Pathological Overall altered	10% (4/40) 7.5% (3/40) 17.5% (7/40)	8.9% (8/90) 4.4% (4/90) 13.3% (12/90)	0.04 0.50 0.38	0.84 0.48 0.53	0.02 0.06 0.05

*Statistically significant (p ≤ 0.05); χ^2^ = chi-square test; p-value of chi-square test; V = Cramer’s V

### School age

Overall, both in the KS group and in the control group, questionnaire scores regarding school-aged subjects were higher than those of preschoolers.

With the exception of the SHY factor, mean T-scores were higher in the KS group compared to the control group, with statistical significance and moderate effect sizes achieved for the DIMS, DA, DOES, and total T-scores (p < 0.01 for all; *r* = 0.31, *r* = 0.30, *r* = 0.41, and *r* = 0.39, respectively) (Table 3).

**Table 3:** School age: differences in total and individual factor T-scores between KS group and control group

	T-score (mean ± standard deviation)		p-value	Effect size
	Cases	Controls		
**Disorders of initiating and maintaining sleep**	58.3 ± 14.1	52.2 ± 10.7	< 0.01 *	0.31
**Sleep breathing disorders**	53.8 ± 10.2	52.6 ± 12.1	0.21	0.13
**Disorders of arousal/nightmares**	56.7 ± 12.5	51.3 ± 10.7	< 0.01 *	0.30
**Sleep–wake transition disorders**	55.1 ± 13.0	51.2 ± 10.3	0.10	0.18
**Disorders of excessive somnolence**	59.1 ± 13.6	51.1 ± 11.8	< 0.01 *	0.41
**Sleep hyperhidrosis**	51.9 ± 10.0	54.2 ± 13.3	0.97	< 0.01
**TOTAL**	60.7 ± 12.4	53.3 ± 11.8	< 0.01 *	0.39

*Statistically significant (p ≤ 0.05). For the Mann–Whitney test, effect size is given by the rank biserial correlation (*r*)

With regard to the percentages of patients with clinically significant T-scores (total and individual factors), statistically significant differences were found for pathological DIMS, borderline DA, overall altered DA, borderline DOES, overall altered DOES, borderline SHY, borderline total, and overall altered total T-scores. They were higher in the KS group compared to controls, with effect sizes indicative of a strong or very strong association (Table 4).

**Table 4:** School age: percentages of subjects with clinically relevant total and individual factor T-scores

		Percentage (n/total)		χ^2^	p-value	V
		Cases	Controls			
**Disorders of initiating and maintaining sleep**	Borderline Pathological Overall altered	27.5% (11/40) 17.5% (7/40) 45% (18/40)	28.9% (26/90) 5.6% (5/90) 34.4% (31/90)	0.02 4.70 1.33	0.87 0.03 * 0.25	0.02 0.29 0.11
**Sleep breathing disorders**	Borderline Pathological Overall altered	25% (10/40) 12.5% (5/40) 37.5% (15/40)	15.6% (14/90) 12.2% (11/90) 27.8% (25/90)	1.58 < 0.01 1.20	0.20 0.96 0.27	0.13 < 0.01 0.11
**Disorders of arousal/nightmares**	Borderline Pathological Overall altered	42.5% (17/40) 7.5% (3/40) 50% (20/40)	14.4% (13/90) 4.4% (4/90) 18.9% (17/90)	17.30 0.47 12.90	< 0.01 * 0.48 < 0.01 *	0.52 0.07 0.33
**Sleep–wake transition disorders**	Borderline Pathological Overall altered	37.5% (15/40) 7.5% (3/40) 45% (18/40)	28.9% (26/90) 3.3% (3/90) 32.2% (29/90)	0.94 1.01 2.20	0.33 0.30 0.16	0.07 0.10 0.11
**Disorders of excessive somnolence**	Borderline Pathological Overall altered	42.5% (17/40) 15% (6/40) 57.5% (23/40)	13.3% (12/90) 7.8% (7/90) 21.1% (19/90)	16.60 1.60 18.00	< 0.01 * 0.21 < 0.01 *	0.50 0.17 0.41
**Sleep hyperhidrosis**	Borderline Pathological Overall altered	40% (16/40) 2.5% (1/40) 42.5% (17/40)	20% (18/90) 13.3% (12/90) 33.3% (30/90)	5.40 3.40 4.60	0.02 * 0.06 0.32	0.25 0.21 0.24
**TOTAL**	Borderline Pathological Overall altered	52.5% (21/40) 15% (6/40) 67.5% (27/40)	26.7% (24/90) 8.9% (8/90) 35.6% (32/90)	18.90 1.50 21.40	< 0.01 * 0,23 < 0.01 *	0.45 0.13 0.45

*Statistically significant (p ≤ 0.05); χ^2^ = chi-square test; p-value of chi-square test; V = Cramer’s V

## Discussion

Our research demonstrates that children with KS exhibit a higher frequency of sleep-related problems compared to their peers in the general population, particularly among school-aged subjects. While sleep disturbances are less prevalent in our preschool-aged group, this developmental period is crucial due to the rapid physical and cognitive growth that occurs, making adequate sleep essential for optimal development. Sleep disorders are common in this age range [19], with prevalence estimates ranging from 14 to 40% in general population, depending on methodologies and cultural factors [20–24]. These disturbances can have long-term adverse effects, particularly if they persist beyond early childhood, as they are linked to behavioral, emotional, and cognitive challenges [25, 26]. Our findings revealed a lower prevalence of sleep disturbances among preschool-aged children with KS compared to general population estimates. This difference may stem from the unique characteristics of the KS cohort or methodological variations, such as reliance on parental reporting or the specific tools employed. Additionally, sociocultural factors and healthcare access in different regions could contribute to discrepancies in prevalence rates across studies.

Notably, in school-aged children with KS, we observed a higher prevalence of borderline and pathological sleep scores compared to preschoolers. This aligns with previous studies indicating that sleep disturbances become more pronounced with age [27]. The higher prevalence in school-aged children with KS was also more significant than that reported in the general population, where rates typically range from 22.6% to 55.5%, depending on the context and assessment tools used [28–31].

The impact of the COVID-19 pandemic on sleep patterns, particularly in children, has been well-documented, and may play a role in sleep disturbances. Bruni et al. (2022) investigated the prevalence of sleep disorders in children and adolescents before and during lockdowns, using a standardized questionnaire similar to the one used in the present study (the SDSC, albeit in a modified version) [32]. The authors reported an increase in specific sleep disturbances, primarily difficulties in falling asleep, bedtime anxiety, nightmares, and night terrors, with younger children being more affected. Adolescents, on the other hand, experienced fewer disruptions, potentially because their sleep–wake aligning better aligned with their physiological needs during lockdowns. The heightened occurrence of insomnia symptoms, nightmares, and night terrors in younger children may be associated with anxiety stemming from the uncertainty surrounding the pandemic, a trend also reported in various studies involving adults [33–35] or high school students [36]. Specifically, the notable increase in the frequency of nightmares, observed across all age groups except adolescents, could be construed as an indicator of acute stress-related symptomatology associated with COVID-19; this interpretation has also been noted in another Italian study involving adult subjects [37]. These findings align with our results, as our data collection occurred after the COVID-19 lockdowns. It is plausible that the effects of the pandemic, including heightened anxiety and disrupted routines, contributed to the elevated T-scores observed in our cohort of school-aged children with KS, particularly in domains such as DIMS, DA, and DOES. Furthermore, the bidirectional relationship between poor sleep quality and anxiety, as reported in various studies [38, 39], may have acted as a factor in exacerbating sleep disturbances in our sample.

The higher prevalence of DIMS, DA, and DOES in our cohort of subjects with KS, especially among school-aged children, may be attributable to other factors beyond the post-pandemic context. DA and DIMS are well-documented comorbidities of anxiety and depression [40], which are common in pediatric patients with KS [9]. These psychiatric conditions frequently contribute to disturbances in sleep patterns, particularly in terms of sleep onset and arousal disorders [41]. In children with KS, these comorbidities may exacerbate sleep problems, leading to more pronounced disturbances. DOES, commonly observed in children and adolescents, pose diagnostic challenges due to the lack of specific and sensitive indicators. They might manifest as irritability, inattention, emotional lability, and hyperactivity [42], which are also hallmark symptoms of attention-deficit hyperactivity disorder (ADHD). Given the higher risk of ADHD in individuals with KS, it is plausible that this neurodevelopmental disorder plays a role in sleep-related challenges [43]. The interplay between impaired sleep, sleepiness, and daytime functioning is often not immediately clear, potentially delaying diagnosis and appropriate treatment [44]. If left untreated or misdiagnosed, excessive daytime sleepiness can lead to inappropriate use of medication, which may worsen sleep-related issues and behavioral problems [45]. Conditions such as sleep deprivation, circadian rhythm disorders, and obstructive sleep apnea (OSA), should also be ruled out in cases of DOES [42]. Obesity is a significant risk factor for OSA, particularly visceral fat rather than subcutaneous or total body fat [46]. However, our cohort did not include overweight or obese participants. Other potential contributing factors may include metabolic abnormalities, such as elevated serum leptin levels, which have been shown to correlate with OSA regardless of body weight [47]. Leptin, a hormone produced by adipose tissue, plays a crucial role in regulating body weight and has been found to be elevated in individuals with KS [48]. While leptin levels were not measured in the present study, further research is warranted to explore its role in sleep disturbances in children with KS. A recent case report has suggested the potential multifactorial nature of the etiology of sleep disturbances within the syndrome, although in an adult subject [49]. The potential roles of OSA, iatrogenic central apneas due to testosterone therapy, and sleep–wake circadian rhythm disorders with altered melatonin secretion have been addressed. The latter condition has previously been documented in patients with KS [50, 51]. While some of these factors may also be relevant for pediatric patients with KS, others are less applicable. For example, testosterone levels are typically normal in children with KS until adolescence [2], and testosterone replacement therapy in this age group is usually reserved for specific indications, such as micropenis in childhood or hypergonadotropic hypogonadism in adolescence [14].

Considering the well-established increased risk of neurodevelopmental and psychopathological disorders in individuals with sex chromosome trisomies [52], the additional X chromosome may be a contributing factor to sleep disturbances. Future research should explore possible correlations between sleep disturbances and hormone levels, particularly testosterone and inhibin B, which are key markers of male gonadal function. Additionally, delving into the role of additional chromosomal material as a potential element in the etiopathogenesis of such disorders is warranted [53].

This study provides a novel and comprehensive evaluation of sleep-related behaviors in children with KS. Unlike prior research, which primarily focused on adults and often limited assessments to overall sleep quality or specific issues like insomnia, our study also investigated other sleep disorders, including parasomnias and excessive daytime sleepiness. Interestingly, school-aged children in our sample exhibited a higher prevalence of clinically relevant SDSC scores compared to preschoolers. This may be attributed to hormonal changes in older children, as well as altered hypothalamic regulation of sleep and arousal in those with KS [54].

Several limitations of this study must be acknowledged. First, data collection was cross-sectional and relied on parental reporting, as the questionnaires assessed certain sleep disturbances and behaviors that children might not be aware of, such as sleepwalking and night terrors. While parental reporting is effective for identifying behavioral and developmental problems [55] as well as sleep disorders [56, 57], it may lack the depth and nuance of direct child self-reports, particularly for older children. Second, neuropsychiatric comorbidities, which could influence sleep in patients with KS, were not systematically assessed. Future studies should incorporate comprehensive evaluations of comorbidities and objective measures such as actigraphy and polysomnography to confirm and expand upon our results. Third, while no subjects in our cohort were classified as overweight or obese, and none had received testosterone therapy, other potential influencing factors warrant exploration. Additionally, it is important to consider that a period of six months may, in some circumstances, be slightly less reliable compared to a shorter observation period. Finally, the SDSC, though widely validated in general pediatric populations, has not been specifically validated for use in children with KS.

## Conclusion

Our study found that sleep disorders are more common in children with KS compared to the general population. Screening for sleep-related problems using tools like the SDSC is warranted in clinical practice. Given the higher prevalence observed in the school-age group, a suitable age to start screening could be from 6 years. Children with KS who present with sleep dysfunction may also benefit from an otolaryngology evaluation to identify and address potential underlying issues. Further research is needed to explore the underlying etiology of sleep disturbances in this population, as well as to investigate the potential role of comorbidities and the long-term trajectory of these disorders. Addressing these factors will be crucial for improving the diagnosis and management strategies of sleep-related issues in children with KS.

## Data Availability

No datasets were generated or analysed during the current study.

## Abbreviations

ADHD: Attention-deficit hyperactivity disorder
DA: Disorders of arousal
DIMS: Disorders of initiating and maintaining sleep
DOES: Disorders of excessive somnolence
KS: Klinefelter syndrome
NRS: Non-restorative sleep
OSA: Obstructive sleep apnea
PAR: Parasomnias
SBD: Sleep breathing disorders
SD: Standard deviation
SDSC: Sleep Disturbance Scale for Children
SHY: Sleep hyperhidrosis
SWTD: Sleep-wake transition disorders
